# Strategies and Perspectives for UV Resonance Raman Applicability in Clinical Analyses of Human Sperm RNA

**DOI:** 10.3390/ijms222313134

**Published:** 2021-12-04

**Authors:** Maria Pachetti, Francesco D’Amico, Luisa Zupin, Stefania Luppi, Monica Martinelli, Sergio Crovella, Giuseppe Ricci, Lorella Pascolo

**Affiliations:** 1 Institute for Maternal and Child Health, IRCCS Burlo Garofolo, 34137 Trieste, Italy; luisa.zupin@burlo.trieste.it (L.Z.); stefania.luppi@burlo.trieste.it (S.L.); monica.martinelli@burlo.trieste.it (M.M.); giuseppe.ricci@burlo.trieste.it (G.R.); lorella.pascolo@burlo.trieste.it (L.P.); 2 Elettra—Sincrotrone Trieste S.C.p.A., SS14—km 163.5, 34149 Trieste, Italy; 3 Department of Biological and Environmental Sciences, College of Arts and Sciences, University of Qatar, P.O. Box 2713, Doha 122104, Qatar; sgrovella@qu.edu.qa; 4 Department of Medical, Surgical, and Health Sciences, University of Trieste, 34149 Trieste, Italy

**Keywords:** UV Resonance Raman spectroscopy, sperm, RNA

## Abstract

Developing a deeper knowledge about the impact of DNA and RNA epigenetic mutations on sperm production and fertilization performance is essential for selecting best quality samples in Assisted Reproductive Technologies (ART). Indeed, sperm RNAs adenine and guanine are likely to be methylated in low quality RNA sperm samples and their study requires the employment of techniques able to isolate high quality nucleic acids. UV resonance Raman spectroscopy represents a valuable tool that is able to monitor peculiar molecular modifications occurring predominantly in nucleic acids, being less sensitive to the presence of other biological compounds. In this work, we used an UV Resonance Raman (UVRR) setup coupled to a synchrotron radiation source tuned at 250 nm, in order to enhance sperm RNAs adenine and guanine vibrational signals, reducing also the impact of a fluorescence background typically occurring at lower energies. Despite that our protocol should be further optimized and further analyses are requested, our results support the concept that UVRR can be applied for setting inexpensive tools to be employed for semen quality assessment in ART.

## 1. Introduction

The detection of specific changes in DNA/RNA sequences is a main challenge both in biology and medicine, with the possibility of revealing cellular states and disease conditions. The applicability of related new methods and concepts to reproductive medicine field is desired, particularly to improve diagnosis and male gamete quality assessment [1,2].

Infertility is a disease of the male or female reproductive system that affects millions of people of reproductive age worldwide. Men infertility affects more than 12% of the whole population and it is considered a multifactorial phenomenon [3,4]. It is widely accepted that medical intervention, i.e., Assisted Reproductive Techniques (ART), could be employed to circumvent male factors to some extent [5,6] and this implies a proper male gamete selection. However, the routinary semen analysis does not investigate possible sperm nucleic acids alteration such as DNA fragmentation, damage or epigenetic modifications (i.e., DNA methylation) [7], which significantly modify the semen quality parameters as well as the fertilization and implantation rates [8]. Some karyotypic abnormalities and mendelian gene mutations are well known to produce sterility. Additionally, some mild to severe infertilities seem to be related to mental stresses, obesity, chemical toxin exposure and many other causes that promote sperm DNA and RNA reshaping, also negatively impacting on DNA and RNA nucleotides, inducing genetic variations or reversible epigenetic changes, whose effects have not been extensively studied yet [9]. To date, considerable attention has been focused on the correlation between DNA methylation and infertility, and some genes have been found to be methylated in patients with an impaired spermatogenesis and/or with reproductive dysfunctions [10], while little is still known about the impact of epigenetic and other modifications of RNA on fertility [11,12].

Despite the role of RNAs and their epigenetic modifications is widely studied in relation to zygote fertilization, metabolic and neurodevelopmental inheritance of the offspring and to embryonic development [11,13,14,15,16,17,18], little is still known about their impacts on sperm functionality and on male fertility. Very recently Qin and colleagues reported that there is an increased population of methylated adenine in boar sperm mRNA during cryopreservation, resulting in altered sperm motility and of the overall quality [19]. Thus, it is possible that in the future, sperm RNA analysis will be a promising diagnostic tool for selecting good quality samples in ART and after sperm cryopreservation; however, this is greatly prevented by the low content of RNA in these cells and the complexity of molecular analysis. In fact, mature spermatozoa are considered as transcriptionally inactive due to the decrement of transcription during the last phases of spermatogenesis. In this process the chromatin is reorganized, and the histone replaced by protamine that densely packed the sperm genome [20]. Nevertheless, a small portion of gene are transcribed and contribute to the sperm transcriptome. Indeed, it has been observed that sperm RNA is mainly localized at nuclear level where the disulfide bridges between protamine tightly packed the nucleic acid making technical difficult their isolation during laboratory extraction procedure [21].

In this picture, tailored and inexpensive approaches like Raman spectroscopy would be highly valuable in the field. As very recently reported by Dai and colleagues, Raman spectroscopy has been listed as a novel and promising technique able to measure DNA modifications such as fragmentation [22]. In fact, Raman spectroscopy is based on the recording of the light diffused inelastically from the sample, and it is able to observe the modification of the vibrational profiles of sample’s molecular components due to the interaction with light. Depending on the molecules investigated and on the extent of the vibrational variation, Raman spectroscopy is able to indirectly provide spectral fingerprints related to the vibrational state of the molecules studied even when samples are in an aqueous solution due to the low Raman cross-section of water bands. To this purpose, D’Amico and colleagues recently exploited the UV Resonance Raman (UVRR) setup and the tunability of the synchrotron radiation source available at Elettra Synchrotron Facility (IUVS beamline) to successfully investigate cytosine methylation pattern of the DNA in model cell lines, which is the most abundant epigenetic change in eukaryotic cells related to several diseases. More specifically, UVRR has been used as a label-free and less time-consuming manner to detect cytosine methylation in cells, tuning the radiation source wavelength to 228 nm in order to selectively and resonantly excite the vibrational modes of cytosine, observing significant vibrational modifications depending on the presence of the methylated form [23]. Working at the same time in resonance condition and in the UV range provide the combined advantages of detecting vibrational modifications in low concentrated samples and to mainly enhance the molecular fingerprints addressed to nucleic acids, respectively.

In the present paper, we describe the experimental strategies adopted to exploit the applicability of UV Resonance Raman set-up coupled with a synchrotron radiation source for prototype RNA analysis. We tuned the synchrotron radiation source to a 250 nm-radiation wavelength in order to investigate the quality of RNA extracted from spermatozoa (derived from 5 patients), compared to RNA isolated from commercial immortalized cell lines, willing to reach the lowest detection limit for RNA and possibly enhancing the spectral regions that could be affected by nucleotide modifications. Additionally, the use of the 250 nm-radiation source is actually a good compromise between the reduction of the fluorescence contribution dominating the Raman spectra at lower energies, where adenine vibrations are resonantly enhanced (i.e., 266 nm) and the relatively good enhancement of adenine peaks occur at 250 nm [23].

## 2. Results

### 2.1. Spermiograms

RNA samples were extracted from five patients, whose characteristics are reported in Table 1. Patients 1, 4 and 5 have a low concentration of spermatozoa (≤15 million/mL, [24]), while total motility parameter classified the patients into 2 subpopulations: patients diagnosed with oligoasthenozoospermia (patients 1, 2 and 5) and with asthenozoospermia (patient 2 and 3).

### 2.2. UV/Vis Absorbance Measurement of RNA

**Figure 1:**
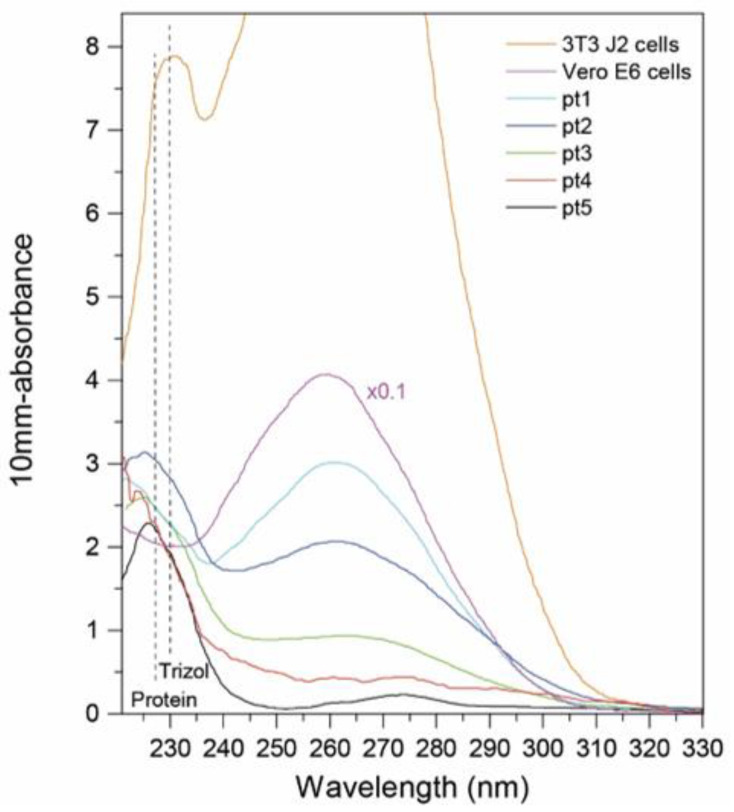
UV-Vis spectra of RNA extracted from 3T3 J2 and Vero E6 cells and from the semen of 5 patients. Vertical dashed black lines indicate the peaks arisen from the presence of proteins and phenolic compounds (i.e., Trizol) in the final eluates.

In Figure 1, the UV/Vis absorbance spectra of model cell lines and 5 patients are presented. With this technique, we calculated the average RNA concentration in the sample, which can be determined by the following formula $c=\frac{A}{\varepsilon l}$, where ε is the molar extinction coefficient, A is the measure absorbance and l is the path length (10 mm in our case). Only in the case of 3T3 J2 and Vero E6 cells, the concentration has not been estimated precisely due to the saturation of the absorption curve. At the same time, by the absorption ratio 260/280 nm, we define the purity of the extracted RNA, which has been analyzed. The presence of chemical contaminants derived from the extraction process is evaluated by the absorption ratio 260/230 nm. The ratio 260/280 nm is approximately 1.8 for the RNA extracted from 3T3, Vero cells and patient 1, while this value is approximately 1.5 for patients 2 and 3 and is lower than 1.5 for patients 4 and 5. In fact, patients 4 and 5 are characterized by UVRR spectra containing a significant protein contribution. Differently, the ratio 260/230 is lower than 2–2.2, indicating a possible chemical contamination of phenols in all the samples, which show an absorption band at 230 nm.

### 2.3. UV Resonance Raman Spectroscopy

**Figure 2:**
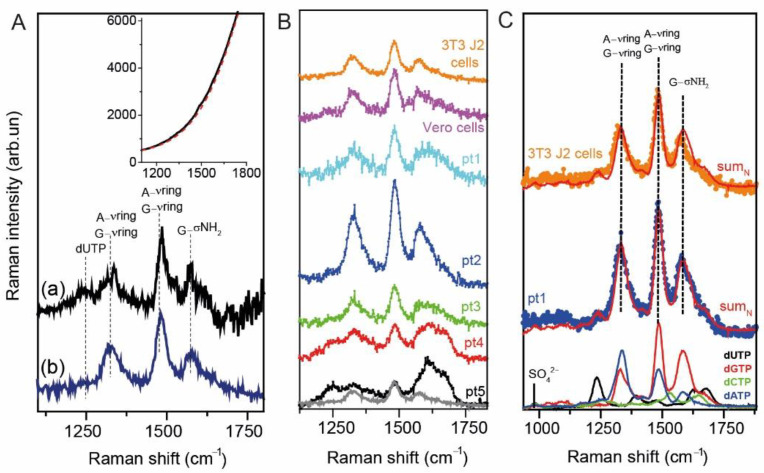
(**A**) The inset shows the UVRR spectra of RNA extracted from the 3T3 J2 cell line (depicted in black) measured using a 266 nm-radiation source. As clearly shown, the spectrum is completely dominated by a fluorescence background, which has been removed following a polynomial baseline (dashed red line). The comparison between the UVRR spectra of the RNA extracted from the 3T3 J2 cell line measured with a (**a**) 266 nm-radiation source (after the baseline removal) and with a (**b**) 250 nm-radiation source have been reported. Both spectra shows that the vibrational features of the samples are maintained, and the only limitation encountered at 266 nm is the dominant contribution of fluorescence. (**B**) UVRR spectra in the range 1100–1800 cm^−1^ of the RNA extracted from 3T3 J2 and Vero E6 cell lines (depicted in orange and purple respectively) and of the RNA extracted by 5 patients. The samples are disposed, going from the highest concentration in the upper part to the lowest concentration in the lower part. (**C**) UVRR spectra of (bottom) dUTP, dGTP, dCTP and dATP solutions probed using a 250 nm-radiation source. The spectra are normalized to the area of the SO_4_^2−^ peak (981 cm^−1^). The sum of the weighted contribution of each nucleotide is reflected in sumN (depicted in red), which has been superimposed to the UVRR spectra of the RNA extracted from patient 1 (in blue) and from the 3T3J2 cell line (depicted in orange).

In order to enhance adenine and guanine contributions and also to avoid the spectral fluorescence background affecting the UVRR spectrum of the nitrogenous bases as previously observed in Reference [23] and furtherly shown in Figure 2A, we chose to work with a 250 nm-radiation source. In fact, as shown in the inset of Figure 2A, the UVRR spectrum of 3T3J2 cells collected using a 266 nm-radiation source is completely dominated by a strong fluorescence background. By removing the background using a polynomial curve (dashed red line in the inset) we obtained the UVRR spectra reported in Figure 2(Aa). The direct comparison between the spectrum of 3T3 J2 cells collected at 266 nm (in black) and at 250 nm (in blue) show no significant spectroscopic differences, with the major advantage that at 250 nm we have not a dominant contribution of fluorescence in the region of interest. Additionally, the 250 nm-radiation is particularly suitable for studying and detecting possible epigenetic modifications such as adenine methylation, typically observed in RNA sequences of spermatozoa by molecular techniques [25,26]. In this light, the use of 250 nm-radiation source for our scopes is reasonable.

Figure 2B shows the UV Resonance Raman spectra of extracted RNA from model cell-lines (3T3 J2 and Vero E6 cells) and from spermatozoa of five enrolled patients. RNA vibrational bands of interest lie in the region 1100–1800 cm^−1^, while the rest of the UVRR spectra are distorted by fluorescence contributions.

In order to estimate the contribution of each nucleotide to the final spectra of extracted RNA, we collected separately also the UVRR spectra of single nucleotides dATP, dGTP, dCTP and dUTP, properly dissolved in a Na_2_SO_4_ aqueous solution (0.2 M). After a proper spectral normalization of the SO_4_^2−^ vibrational peak, located at 981 cm^−1^, it was possible to get a correct estimation of the contribution of each single nitrogenous base contributes to the overall RNA spectral profile (see Figure 2C).

As shown in Figure 2B, the UVRR spectra of the extracted RNAs are disposed vertically by decreasing the concentration of the nucleic acid, i.e., from patient 1 to 5. The contribution of the buffer solution containing RNAse inhibitor has been removed by all the UVRR RNA spectra, after normalizing them to the 3300 cm^−1^ band of the buffer, addressed to the OH-stretching. Interestingly, the UVRR spectra of the RNAse inhibitor does not give any significant contribution in the region of interest and can be easily removed. As reported in Figure 2B,C, all the RNA spectra are characterized by three main peaks located at 1323, 1485 and 1579 cm^−1^, addressed to guanine vibrations, to adenine and guanine stretching/planar ring vibration, and to adenine/guanine ring stretching, respectively [27,28,29,30]. A little band at 1240 cm^−1^ is present and it is addressed to a sum of cytosine, adenine and uracil contributions [30]. Experimental UVRR spectra are matched by an expected RNA spectrum, obtained performing the sum of the UVRR profile of each nitrogenous base are reported in Figure 2C). Thus, we calculated the sum_N_ curve as:

sum_N_ = a ∗ dATP + b ∗ dGTP + c ∗ dCTP + d ∗ dUTP.

**Figure 3:**
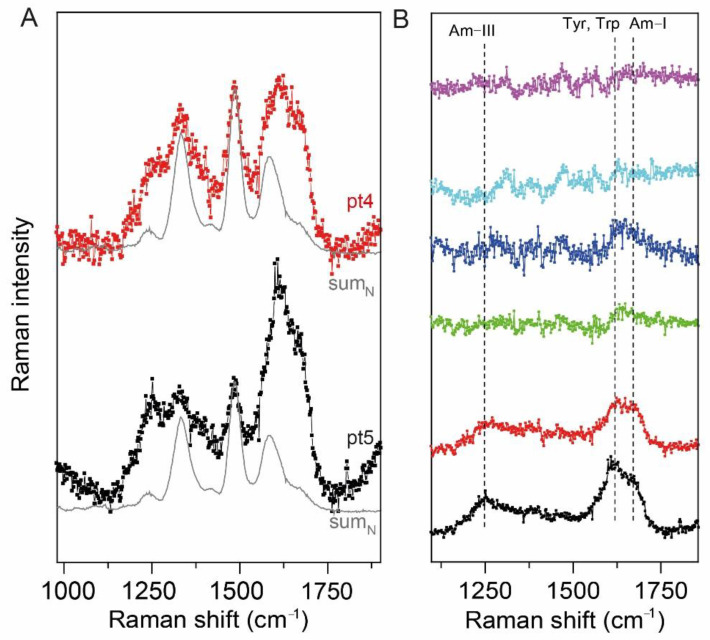
(**A**) UVRR spectra of patients 4 and 5 (depicted in red and black lines + symbol) superimposed by sum_N_ (depicted in gray). In this case, the experimental UVRR spectra are not properly matched by the sum_N_ spectrum. (**B**) the difference UVRR spectra obtained by subtracting the pure spectrum of RNA from the ones collected, in order to enhance the contribution of biological contaminants (i.e., proteins) to the final UVRR spectra.

As clearly shown in Figure 2C, the sum_N_ matches well the experimental UVRR spectra of RNA extracted from model cell lines and patient 1, while it does not properly reproduce the spectra of patients with a 260/280 absorption ratio lower than 1.8 (especially, patients 4 and 5) (see Figure 3A). In fact, as reported in Figure 3A, patients’ 4 and 5 UVRR experimental spectra present a different line shape of the bands compared to the expected RNA spectrum (i.e., sum_N_), which appear broader and formed by other sub-peaks. In order to clean up the UVRR spectra of patients 2 to 5 and identify which is the contaminant responsible for those spectral contributions, we subtracted the spectrum of sum_N_ from the UVRR spectra of the patients (see Figure 3B).

In Figure 3B it is clearly shown that the UVRR spectra of patients 2–5 are affected by the presence of contaminants in the final eluates, characterized by vibrational bands located at 1269, 1605–1615 and 1674 cm^−1^. Interestingly, those bands could be associated with the presence of proteins and, in particular, to the chemical vibrations of amide III, tyrosine and tryptophan residues and amide I contributions, respectively [31,32,33]. 

**Figure 4:**
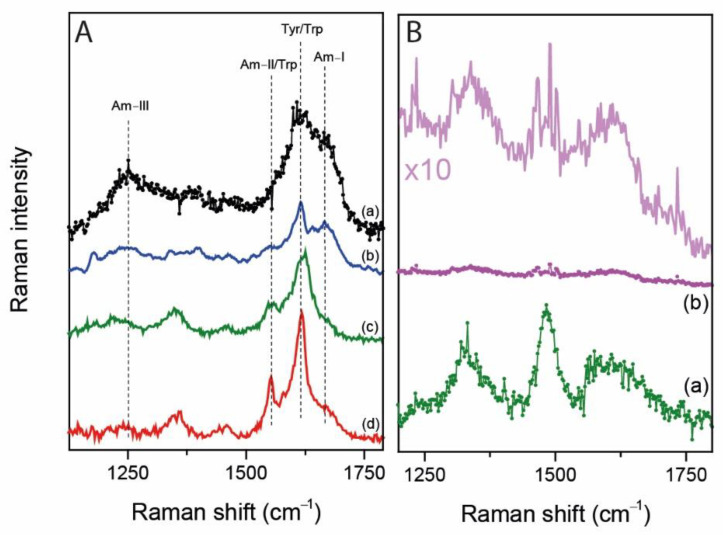
(**A**) (**a**) the 250 nm UVRR spectrum of patient 5 with the pure RNA contribution removed is compared with the previously published 244 nm-UVRR spectra of different proteins such as (**b**) α-synuclein fibrils (unpublished data), (**c**) lysozyme fibrils and (**d**) freshly prepared solution of lysozyme [31]. Typical proteins’ contributions are highlighted with dashed black lines. (**B**) UVRR spectra of patient 3 collected with (**a**) and without (**b**) the presence of the RNAse inhibitor (in green and in magenta, respectively). In order to compare both spectra (**a**,**b**), the spectrum of (**b**) has been multiplied by a factor 10.

Because of that, in Figure 4A we reported the UVRR spectra of different proteins, as natively folded and fibrillated forms, recently collected during previous beamtimes using the 244 nm-radiation source in comparison with the difference spectrum of patients 5. Indeed, we chose to present patient 5 because of the presence of contaminants is maximally visible in this sample having the lowest concentration of the RNA in the study. Due to the heterogeneity of proteins present in spermatozoa, especially in terms of their native secondary structures, and also of proteins’ structural changes possibly induced by the RNA extraction protocol, we reported the UVRR spectra of α-synuclein (fibrils) and of lysozyme (fibrils and soluble natively folded). Interestingly, the proteins’ vibrational features, especially those of fibrils, match quite well to the difference spectrum of patient 5 (see Figure 4A). The increased spectral contamination by proteins is inversely proportional to the RNA concentration in the samples as reported in Table 2. Being prevalent present in the final eluates, the contribution of proteins starts to dominate the UVRR spectra when the RNA concentration is very low, despite nucleotides vibrational modes are strongly resonantly enhanced. The consequence is the appearance of proteins-related bands mixed with the nucleotides vibrational peaks.

Consequently, we observed that when the RNA concentration is more than 100–120 ng/µL and the 260/280 ratio is higher than 1.8, RNA vibrational bands are those maximally enhanced by the incoming energy, and the proteins’ contribution (if present) is really negligeable (non-resonantly enhanced). At RNA concentrations below this threshold, if the 260/280 ratio is lower than 1.8, the spectral proteins’ contribution becomes relevant.

Apart from the presence of biological contaminants, the UVRR spectra collected have been obtained after a careful optimization of the measurement protocols. For our scope and due to the Raman setting, we found out that the use of a RNAse inhibitor is mandatory to prevent the gradual enzymatic degradation of the RNA during the measurement, which leads to a lower signal-to-noise ratio. In fact, in Figure 4B the spectra of patient 3 RNA with and without inhibitor have been reported.

## 3. Discussion

In medicine, the damages and methylations of nitrogen bases are now of increasing interest when specifically affecting RNA, and the translational potential of Raman spectroscopy is in this field is still poorly investigated. We already reported that UV Resonance Raman spectroscopy represents a valuable tool for studying DNA damages [1] and its epigenetic modifications such as cytosine methylation, which can lead to the development of several diseases [23]. Based on this, in this work, we specifically evaluated the applicability of UVRR to study the RNA extracted from spermatozoa for a future possible application in reproductive medicine. Among cells, spermatozoa are very tricky and challenging ones for Raman analyses since the RNA extraction procedure is trivial, requiring time and adjustments in order to increase the yield and the quality. Noteworthy, compared to most model cell lines, the quantity of extracted RNA from semen samples is very low, magnifying the effect of possible biological and chemical contamination that could lead to misleading interpretation in the analysis of the spectra. Additionally, RNA is not stable in general for long measurements and the presence of an RNAse inhibitor is thus mandatory for our purposes (Figure 4B) to avoid enzymatic digestion and RNA degradation over time.

Our Raman measurements were performed not only taking into account both the enzymatic degradation, but also the potential photodegradation induced by the incoming radiation. Thus, a preliminary optimization of the radiation power from the synchrotron source and of the rotating sample holder has been made prior to starting.

As already mentioned, the Raman spectra of nucleotides and RNA measured using a 266 nm-radiation source are dominated by a strong fluorescence background that can be reduced by choosing a higher-energy radiation wavelength such as 250 nm. Noteworthily, working at 250 nm represents the best choice in order to enhance also the vibrational modes of adenine as observed from the nucleotides spectra, which is one of the most abundant methylated nucleotides in spermatozoa [34].

The spectra of RNA can be easily recorded in the case of RNA extracted from 3T3 J2 and Vero E6 cell lines, since a huge amount of nucleic acid can be purified, ensuring a good signal-to-noise ratio of the spectra. The resonant condition assures a minimization of the spectra alterations caused by biological and chemical contaminants, such as proteins and phenols, since the vibrational contributions of RNA are selectively and sufficiently enhanced. Differently, in the case of RNA extracted from spermatozoa, the quality of the spectra is reduced mainly because of the low concentration. In fact, except for patient 1, we observed that the UVRR spectra of the RNA extracted from patients 2 to 5 are disturbed by other components and the vibrational line shapes of RNA typical peaks become broader. Simulating the subtraction of the sum_N_ spectrum from the UVRR spectra of the patients highlights that those with a RNA concentration lower than 100–120 ng/µL (i.e., patients 2 to 5) contains contributions arisen from the presence of proteins-related bands and, in particular, amide I, amide III and aromatic amino acids ones (see Figure 4A). With this inspection, we found a preliminary threshold concentration over which RNA can be measured with a high degree of spectroscopic purity. Interestingly, the presence of proteins in the final eluates was anticipated by the absorption 260/280 nm ratio calculated from the UV-Vis spectra of the samples. However, despite the presence of proteins, this UVRR setup is able to measure and detect the presence of RNA in the eluate down to 5.3 ng/µL.

In light of these findings, the removal of proteins in the final eluates could improve the sensibility of the UVRR technique, thus decreasing the Raman volume scattering, allowing the detection of possible nucleic acid modifications up to concentrations lower than those probed in this study. As already discussed in the scientific literature and in our previous studies, UVRR is able to detect spectral modifications induced by changes in the nitrogenous bases chemical constitution [23]. This effect is suitable, for example, to detect epigenetic disorder induced by methylated nitrogenous bases in RNA [35].

In this regard, on the basis of the current scientific literature, we expect spectral variations of both adenine and cytosine in the 1300–1650 cm^−1^ wavenumber range. Due to the high sensitivity of UVRR to the nitrogenous bases normal modes, as already demonstrated for DNA [23], we expect that even small modifications in the RNA Raman profile induced by adenine methylation may be detectable and estimated employing UVRR at 250 nm.

Thus, these evidences are put forward to further explore the topic and to possibly reveal particularly adenine epigenetic modifications in clinical samples, such as N1 mA and N6 mA which are commonly found in RNA. Conversely, the use of a 250 nm-radiation source is not optimal for investigating cytosine methylation since the cytosine contribution to the overall RNA spectra at an excitation wavelength of 250 nm is negligible, similarly to those occurs with DNA samples [23]. To our knowledge, other authors have performed SERS experiments on methylated nitrogenous bases (methyladenine and methylcitosine) [36,37] that evidenced clear changes in the Raman spectral profiles comparing with the pristine bases, and consisting in large modifications in peaks positions and intensities. Such changes have been reported to occur also in the UVRR spectral profiles [38,39] and are thus expected in the spectra of methylated nucleotides, despite not already reported yet in the literature. In this case, it is important to remark that the resonance effect occurring in real UVRR may lead to further unpredictable spectral modifications due to the different micro-environments affecting in a selective way the cross section [31], and thus the intensities and the line shapes of the Raman profile [23,40,41].

## 4. Conclusions

In the present paper we successfully probed the vibrational signatures of RNA extracted from commercial cell lines and spermatozoa from five patients waiting for medical intervention (ART). The use of the 250 nm-radiation source and a proper data reduction allows the analysis of RNA samples without the interference of a strong fluorescence background, while satisfactorily enhancing adenine and guanine vibrational signals. Perhaps Raman spectroscopy has been already indicated as a potential non-invasive technique to measure DNA modifications such as DNA fragmentation in spermatozoa, the present research is the first, to our knowledge, that put the technical basis for future analyses of RNA modifications in sperm. We successfully proved that clear UVRR spectra of the RNA can be obtained only by adding an RNAse inhibitor in the final eluates. This in fact reduces the possibility of chemical degradation, ensuring long-term stability of the nucleic acids throughout the measurements without being an element of spectroscopical disturbance. The spectra are affected by a component deriving from the protein contamination of the samples, and that reassemble mostly misfolded molecules. Despite the presence of proteins and low RNA concentration, that are expectable in clinical samples, the resonant condition and the synchrotron source allowed to successfully identify a spectral region where RNA modifications or methylation should be detectable. Further real analyses are however necessary, possibly in sets of samples with proper molecular characterization, to better demonstrate the potential clinical applicability of this vibrational approach.

## 5. Materials and Methods

### 5.1. Subjects and Sample Preparation

Five male Caucasian infertile patients were enrolled at the assisted medical procreation center of the Institute for Maternal and Child Health IRCCS Burlo Garofolo (Trieste, Italy). The semen samples were collected through masturbation, and after liquefaction, they are observed under an optical microscope to determine the concentration and the motility [24]. All the procedures were conducted following the guidelines of the Declaration of Helsinki (7th version 2013), after the approval by the FVG regional ethics committee (5 mille15D1).

Five hundred microliters of each semen sample were used for the analysis. After two washing in physiologic solution, the sperm pellet was lysed in Trizol reagent (Euroclone, Pero, Italy) and the RNA was extracted following manufacturer’s instruction and eluted in 20 μL of distilled water.

The RNA was also isolated from VeroE6 (epithelial kidney normal cell line from Cercopithecus aethiops, ATCC CRL-1586), and 3T3 J2 (normal embryo fibroblast from Swiss mus musculus) cell lines. After culturing them for 4 days in T75 flasks (about four-million cells) they were washed in phosphate buffer saline solution, and then detached from the bottom of the flask with a cellular scratcher. After centrifugation, the RNA was extracted from the pellet with the protocol employed for the sperm and resuspended in 50 μL of distilled water. The RNA concentration was measured with a Nanodrop instrument (Thermo Fisher Scientific, San Diego, CA, USA).

### 5.2. UV Resonance Raman Spectroscopy Measurements

Ten microliters of extracted RNA solution from semen samples and model cell lines were measured by drop-casting onto a rotating horizontally-disposed sample holder. Measurements were carried out at Elettra Synchrotron Radiation facility, using an experimental setup described elsewhere [42]. A 250 nm incoming radiation wavelength was used to excite the samples with a power of 32 μW. The Raman radiation was collected in backscattering condition. In order to avoid enzymatic and photodegradation, a RNAse inhibitor (20 U in each sample, N8080119, Applied Biosystems, Thermo Fisher Scientific, Waltham, MA, USA) was added in the sample solutions. The Raman instrument was composed of a Czerny-Turner spectrometer with focal length of 750 mm, a holographic reflection grating of 1800 g/mm and a Peltier-cooled back-thinned CCD. Raman frequencies were calibrated on cyclohexane spectra and the spectral resolution was 25 cm^−1^. Final spectra were obtained by averaging 15 spectra of 15 min, for a total integration time of 13,500 s. To obtain the sample spectra, we removed the spectra of the substrate and of the RNAse inhibitor from the samples’ ones.

## Figures and Tables

**Table 1:** Semen parameters (concentration, total and progressive motility and the WHO classification) of the samples investigated in the present study.

Patient	Concentration (10^6^/mL)	Total Motility (%)	Progressive Motility (%)	WHO Classification
1	9	40	30	oligoasthenozoospermia
2	60	50	20	asthenozoospermia
3	28	70	30	asthenozoospermia
4	15	45	25	oligoasthenozoospermia
5	3	35	10	oligoasthenozoospermia

**Table 2:** Estimation of the RNA concentration from model cell lines and from the semen of five patients.

Samples	RNA Concentration (ng/µL)	Volume of Elution (µL)
3T3 J2 cells	>4271	50
VeroE6 cells	1622.5	50
1	120.7	15
2	82.5	15
3	36.6	15
4	16.7	15
5	5.3	15

## Data Availability

Not applicable.
